# Supplementary material for: Improving Catalytic Activity of “Janus” MoSSe Based on Surface Interface Regulation
Source: Molecules. 2022 Sep 16;27(18):6038. doi: 10.3390/molecules27186038 (PMC9506453; doi:10.3390/molecules27186038)
Supplement: Supplementary file 1 [file molecules-27-06038-s001.zip › molecules-1914505-supplementary.pdf]

Supporting Information

Improving catalytic activity of "Janus" MoSSe based on surface interface regulation

Mingqian Wang [a, b,1], Xin Wang [b, 1], Ming Zheng [b], Xin Zhou [b, *]

a Public Teaching Department, Heilongjiang Institute of Construction Technology, Harbin, PR China

b MIIT Key Laboratory of Critical Materials Technology for New Energy Conversion and Storage, School of Chemistry and Chemical Engineering, Harbin Institute of Technology, Harbin, PR China

* Corresponding author.

E-mail address: zhoux@hit.edu.cn

[1] The authors contributed equally to this work.

1. Calculation method of HER activity

$$H^+ + e^- + * \xrightarrow{\Delta G_H} H^*$$

The * represents the active site and H* represents the adsorbed intermediate. According to the computational hydrogen electrode model, ΔG_H can be calculated according to the following relationship:

$$\Delta G_H = \Delta E_H + \Delta E_{ZPE} - T\Delta S_H$$

ΔE_H is the adsorption energy of hydrogen calculated by DFT, ΔE_{ZPE} is the difference between the zero-point energy of adsorbed hydrogen and gas phase hydrogen, T is the temperature (298.15 K), and ΔS_H is the change of entropy between the adsorption state and the gas phase. The entropy of the gas phase molecule is obtained by the NIST database, and the entropy and zero-point energy of the adsorbate can be obtained by calculating the vibration frequency where the entropy value is calculated by the following formula:

$$S(T) = \sum_{i=1}^{3N}\left[-R\ln\left(1 - e^{-\frac{hv_i}{k_B T}}\right) + \frac{N_A h v_i e^{-hv_{i/k_B T}}}{T - e^{-hv_{i/k_B T}}}\right]$$

Where R stands for the ideal gas constant, k_B is the Boltzmann constant, h is Planck constant, N_A is the Avogadro constant, νi represents the vibration of the normal mode, and N represents the number of adsorbed atoms.

2. Calculation method of OER activity

At pH=0, the OER usually goes through the following four steps:

$$\mathrm{H_2O}(l) + * \xrightarrow{\Delta G_1} \mathrm{OH}^* + \mathrm{H}^+ + e^-$$

$$\mathrm{OH}^* \xrightarrow{\Delta G_2} \mathrm{O}^* + \mathrm{H}^+ + e^-$$

$$\mathrm{O}^* + \mathrm{H_2O}(l) \xrightarrow{\Delta G_3} \mathrm{OOH}^* + \mathrm{H}^+ + \mathrm{e}^-$$

$$\mathrm{OOH}^* \xrightarrow{\Delta G_4} * + \mathrm{O_2}(g) + \mathrm{H}^+ + e^-$$

The change in free energy is calculated by the following formula:

$$\Delta G = \Delta E + \Delta E_{ZPE} - T\Delta S + \Delta G_U + \Delta G_{pH}$$

ΔE refers to the energy obtained by DFT calculation; $\Delta G_U = -eU$, where U is the electrode potential and e is the number of transferred electrons; $\Delta G_{pH} = k_B T \ln 10 \times pH$ in this work, pH = 0 was employed. A method developed by Nørskov et al was employed to calculate OER overpotentials, using the developed method of Nørskov et al:

$$\eta^{OER} = \frac{\max\{\Delta G_1, \Delta G_2, \Delta G_3, \Delta G_4\}}{e} - 1.23\ \mathrm{V}$$

3. HER and OER activity of Ni_4@MoSSe

The stability of monatomic catalysts is a very important problem. Because of the high surface free energy, isolated metal atoms can easily gather into clusters. Therefore, the OER and HER activities of MoSSe-loaded Ni clusters were further calculated by taking Ni4 clusters (the smallest stereoscopic clusters with the smallest Ni) as an example, as shown in Figure S7.

Free energy changes are shown in Figure S8, compared with Ni@MoSSe, the

overpotential value of OER increased by 0.30 V, respectively. Surprisingly, the overpotential value of HER decreased by 0.54 V, as shown in Figure S9. The reason may be that after Ni aggregates into small clusters, MoSSe acts as the base material to enhance the electrical conductivity, and the Ni atom acts as an excellent HER electrocatalyst in synergy with MoSSe, and the performance is significantly improved Therefore, we can conclude that Ni@MoSSe can maintain good OER and HER activities even if isolated Ni atoms are clustered into small clusters.

4. Figures

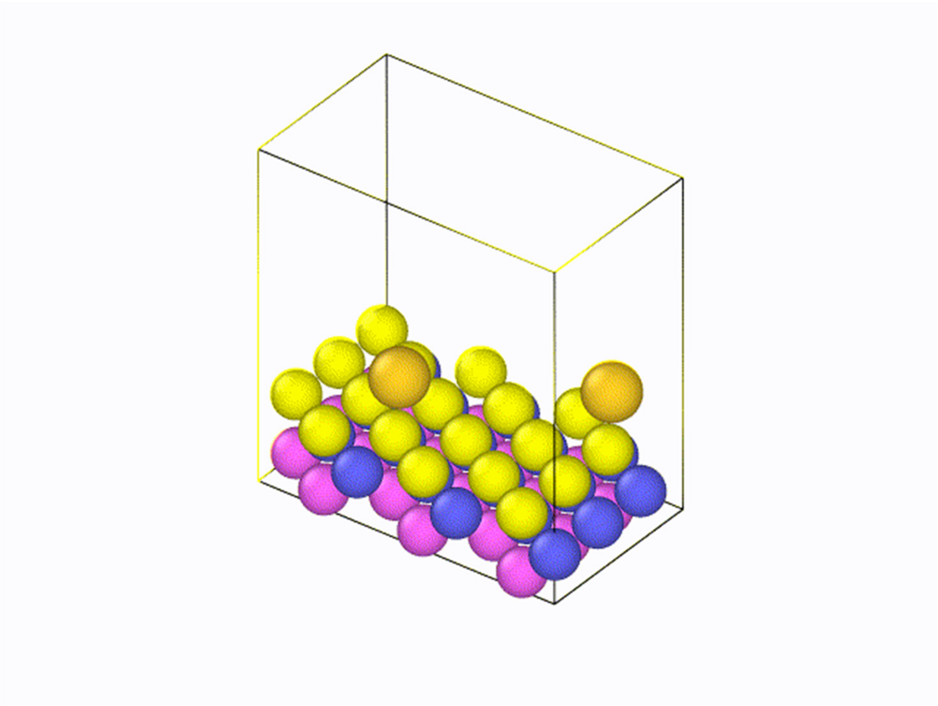

Figure S1 The Au@MoSSe trajectories of AIMD simulation every 10th frame. The simulation is run under 100 to 500K for 3ps with a time step of 1 fs.

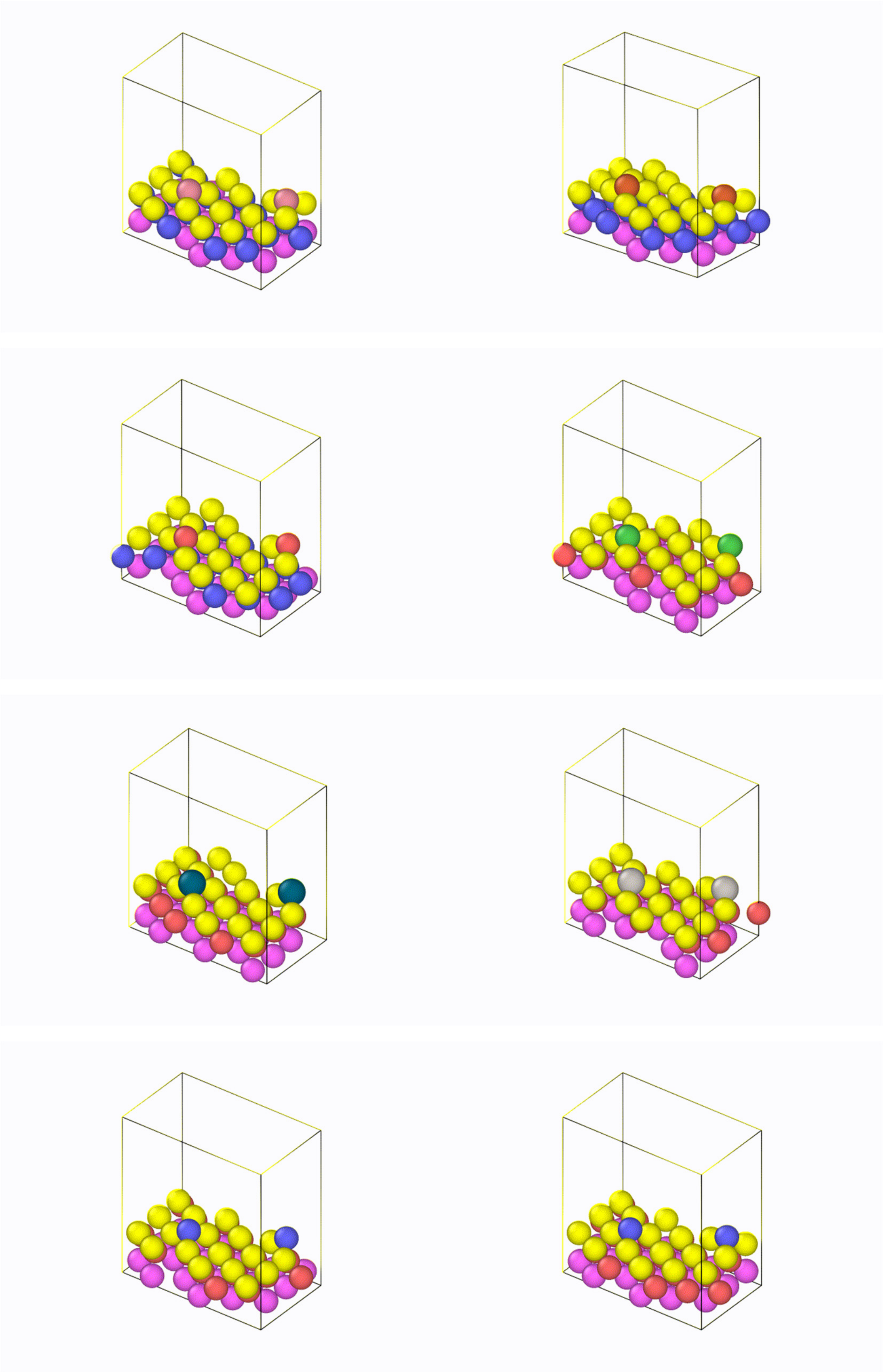

Figure S2 The TM@MoSSe (TM =Co, Fe, Ir, Ni, Pd, Pt, Rh and Ru) trajectories of AIMD simulation every 20th frame. The simulation is run under 500K for 10ps with a time step of 1 fs.

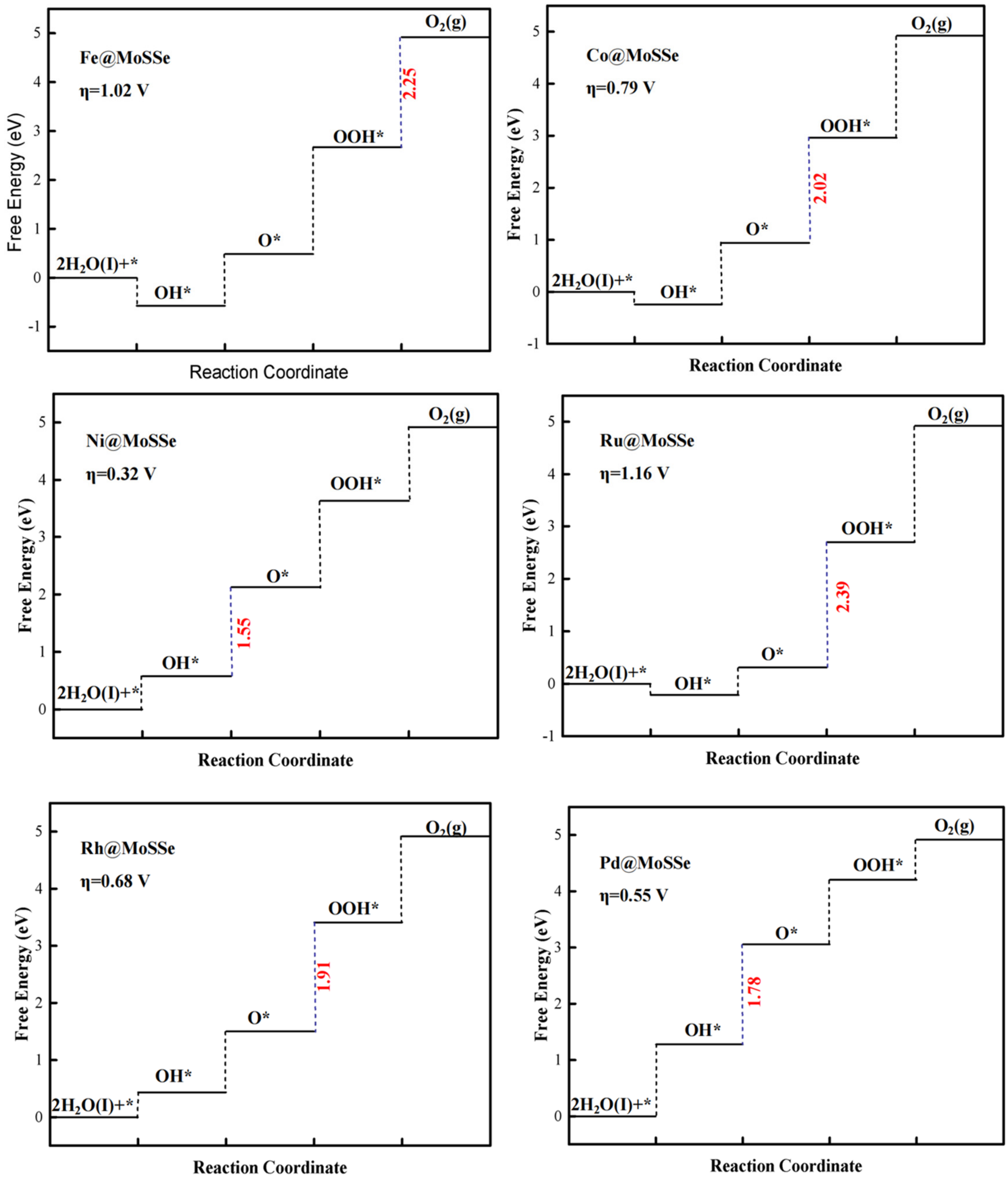
Fe@MoSSe
η=1.02 V
Free Energy (eV)
Reaction Coordinate
2H2O(l)+*
OH*
O*
OOH*
O2(g)
2.25
Co@MoSSe
η=0.79 V
Free Energy (eV)
Reaction Coordinate
2H2O(l)+*
OH*
O*
OOH*
O2(g)
2.02
Ni@MoSSe
η=0.32 V
Free Energy (eV)
Reaction Coordinate
2H2O(l)+*
OH*
O*
OOH*
O2(g)
1.55
Ru@MoSSe
η=1.16 V
Free Energy (eV)
Reaction Coordinate
2H2O(l)+*
OH*
O*
OOH*
O2(g)
2.39
Rh@MoSSe
η=0.68 V
Free Energy (eV)
Reaction Coordinate
2H2O(l)+*
OH*
O*
OOH*
O2(g)
1.91
Pd@MoSSe
η=0.55 V
Free Energy (eV)
Reaction Coordinate
2H2O(l)+*
OH*
O*
OOH*
O2(g)
1.78

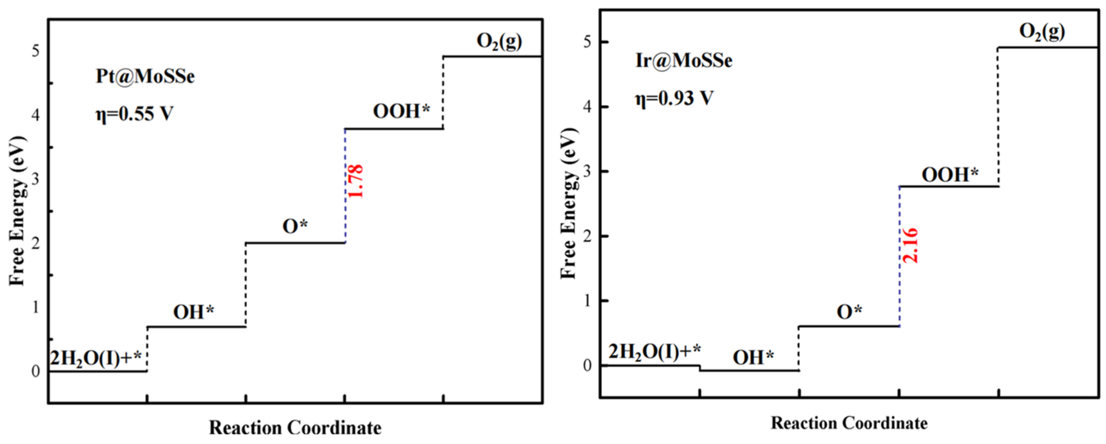

Figure S3 Free energy diagram for the OER of the TM@MoSSe at an electrode potential of U=0 V

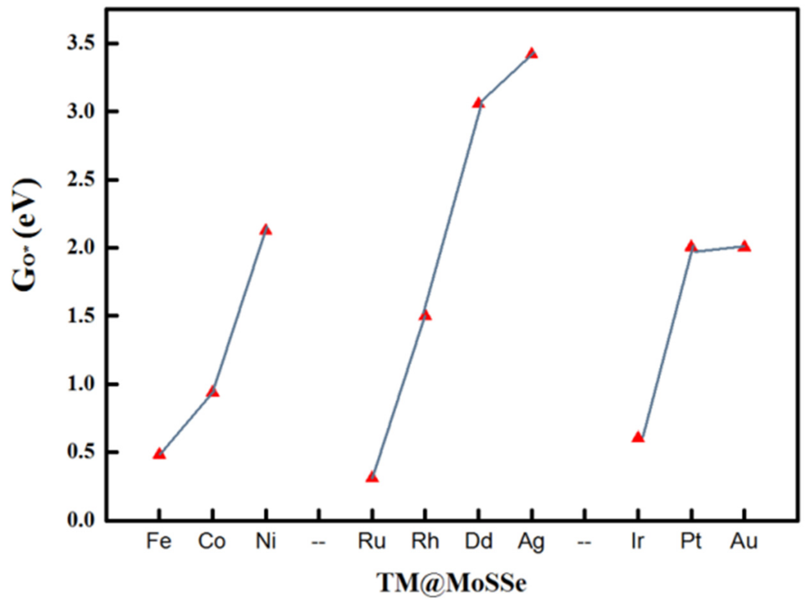

Figure S4 Gibbs free energy diagram of the adsorption intermediates O*.

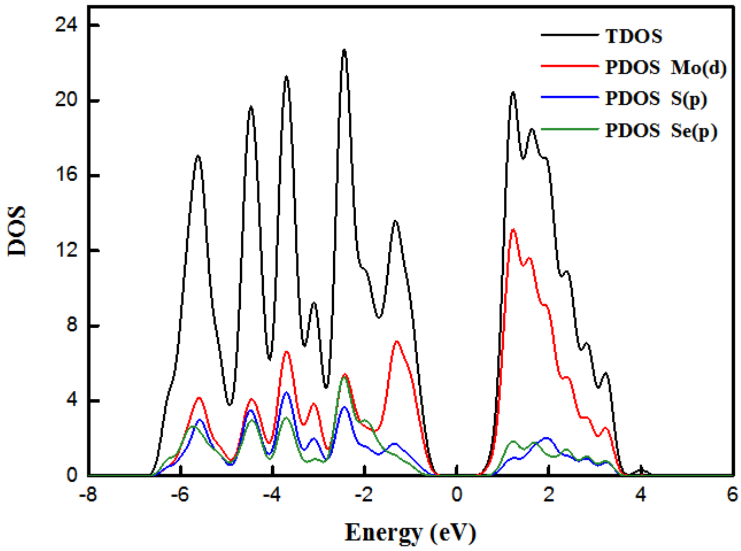

Figure S5 Density of states of MoSSe.

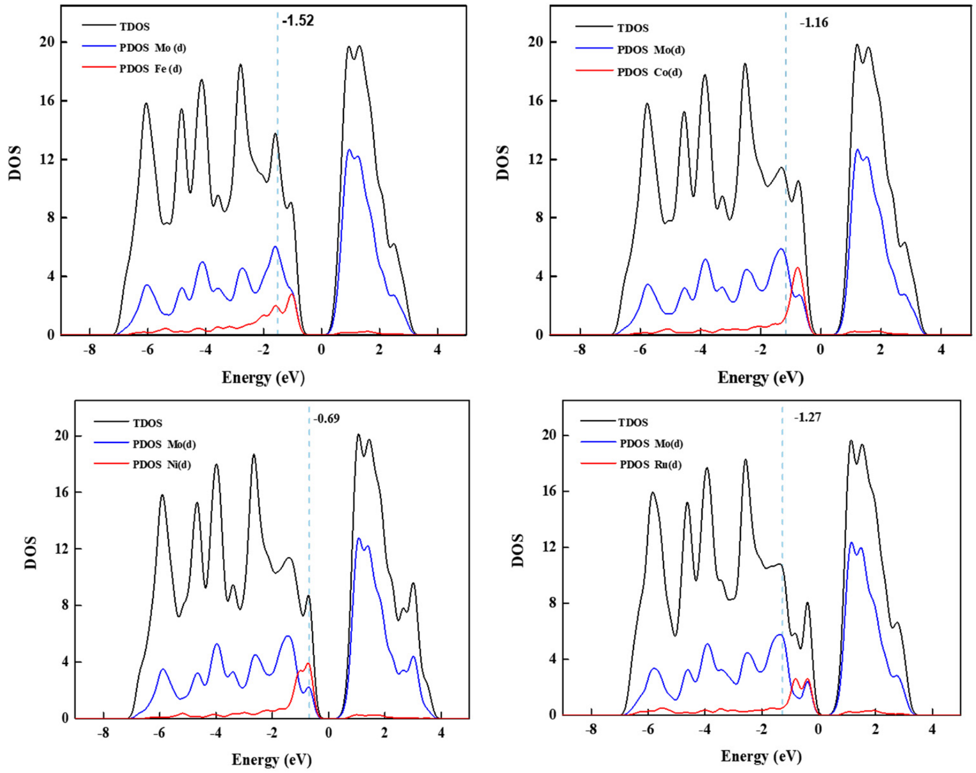

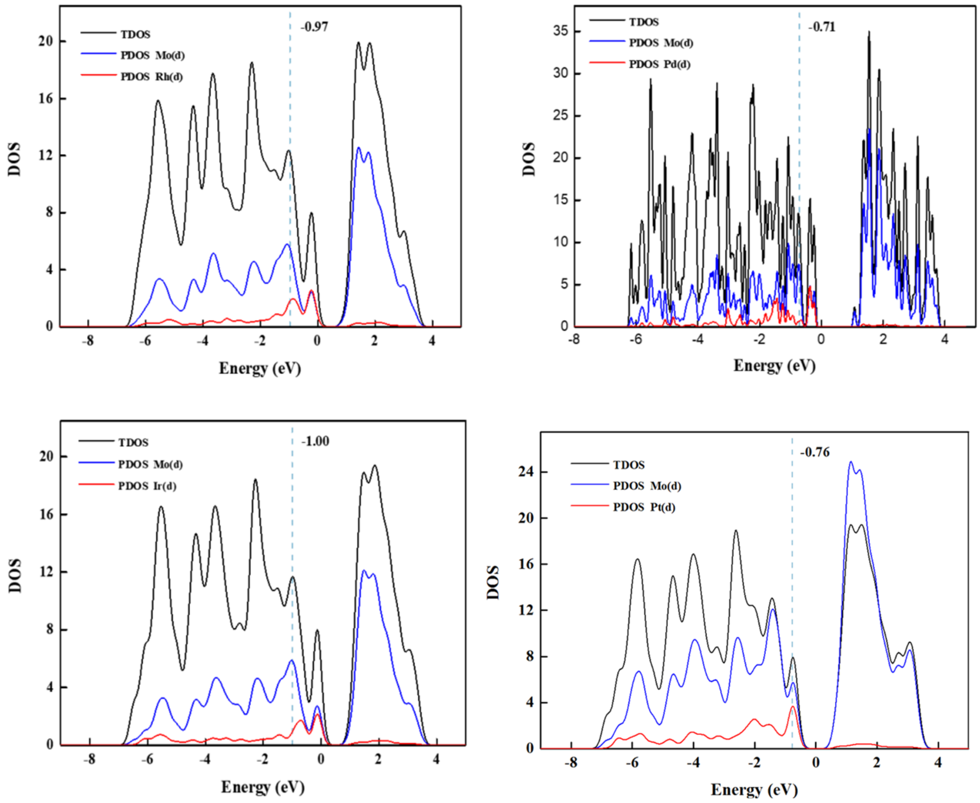

Figure S6. Calculated DOS and d band center of the TM atoms of TM@MoSSe.

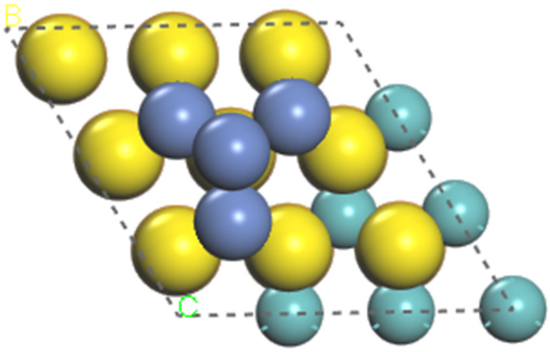

Figure S7 The structure of the Ni4@MoSSe.

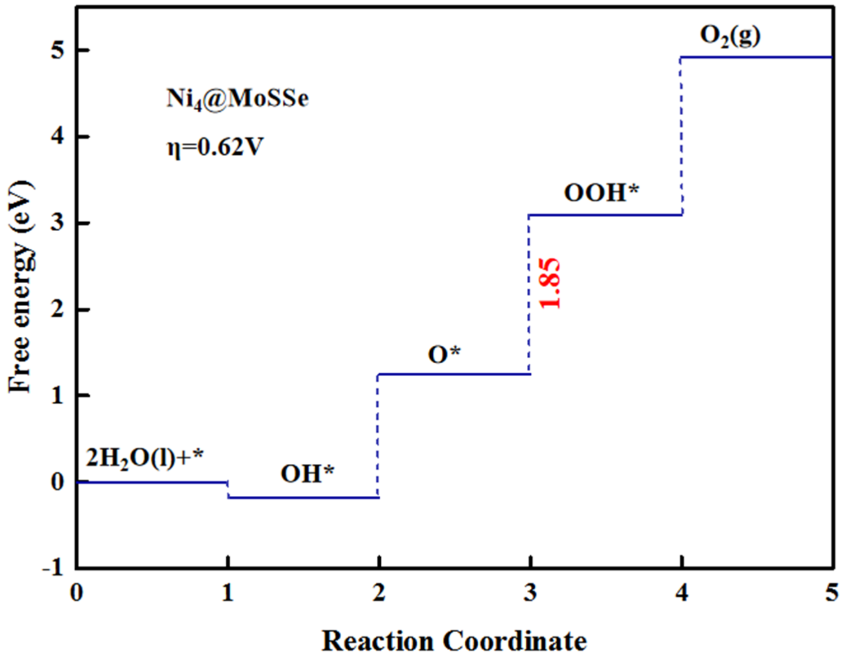

Figure S8 Free energy diagram for the OER on Ni4@MoSSe.

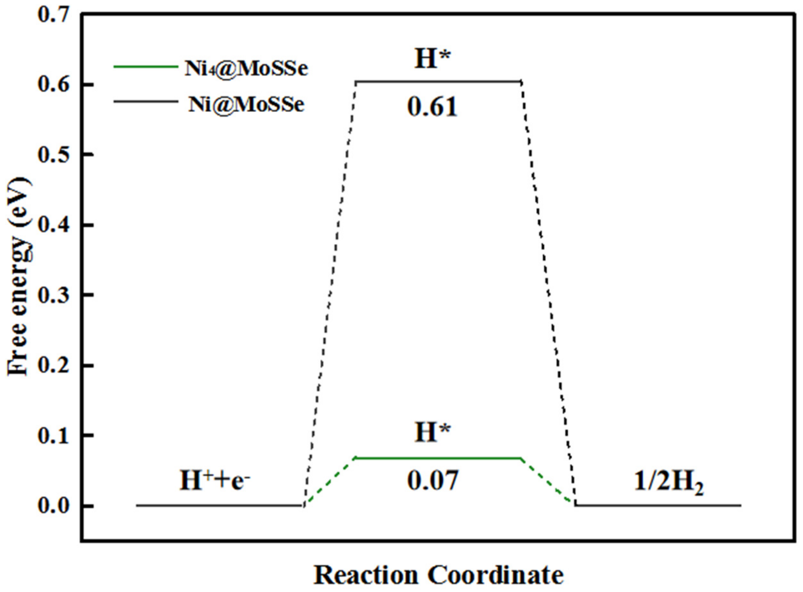

Figure S9 The Gibbs free energy of Ni4@MoSSe.

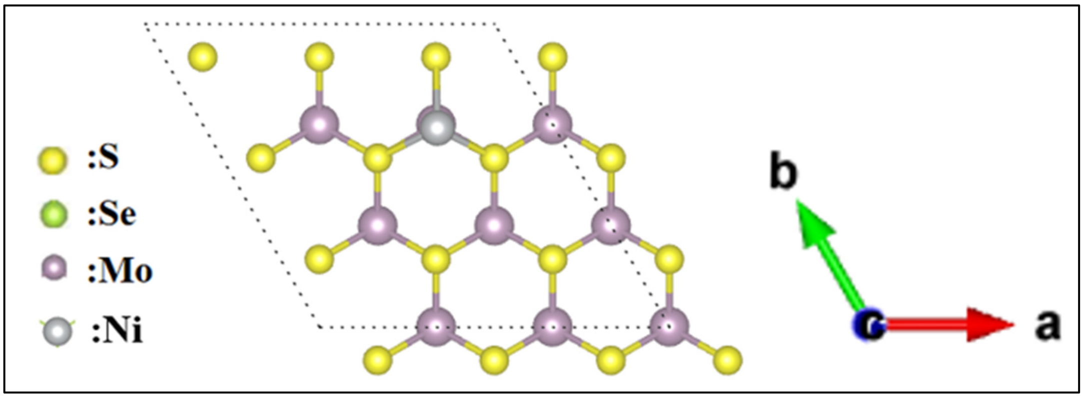

Figure S10 The strain is applied in the A and B directions of Ni@MoSSe.

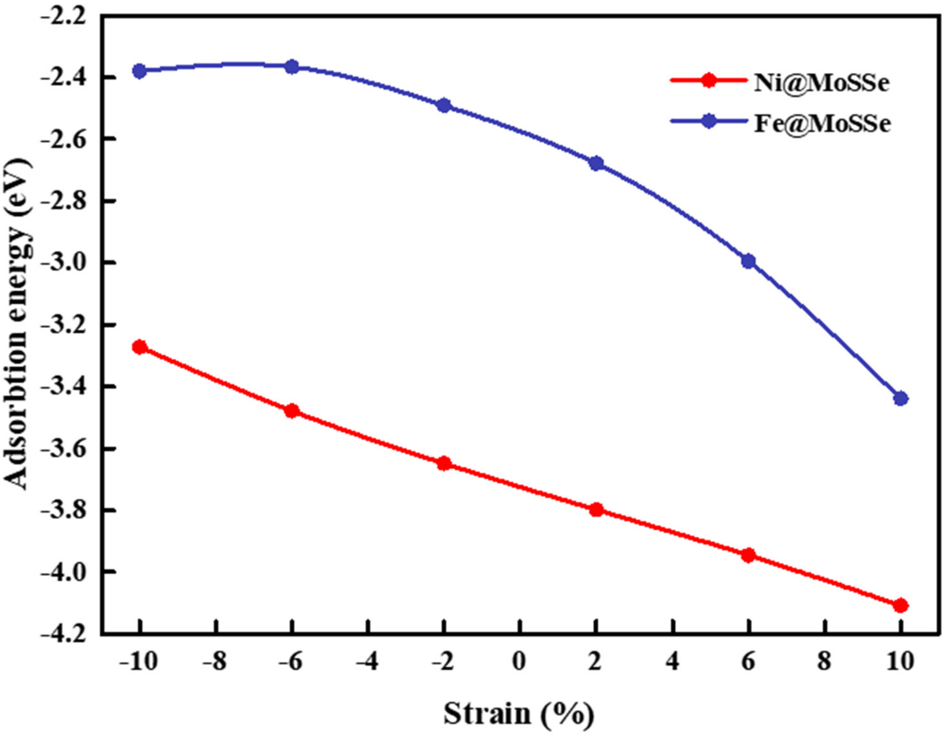

Figure S11 Curve of Ni@MoSSe and Fe@MoSSe adsorption energy as a function of strain.

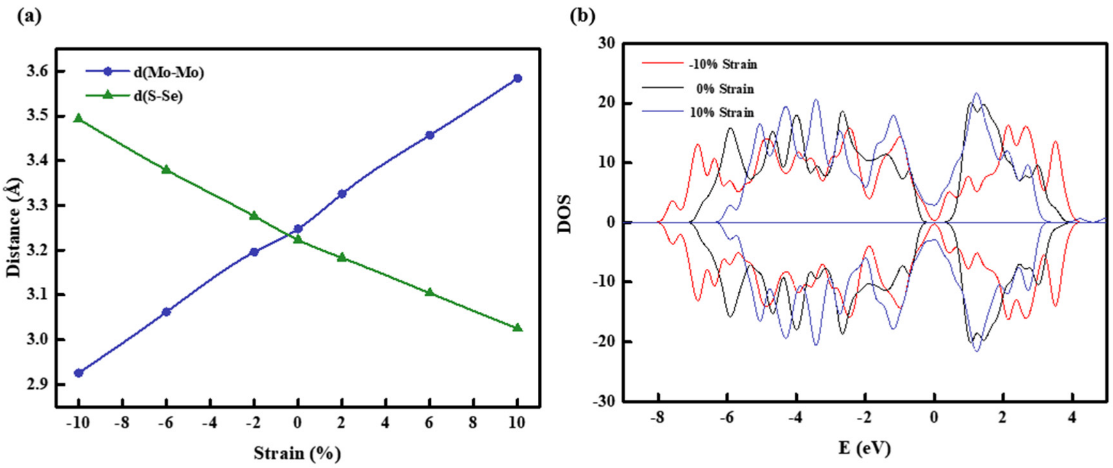

Figure S12 Effect of strain on Ni@MoSSe (a) structure and (b) density of state.

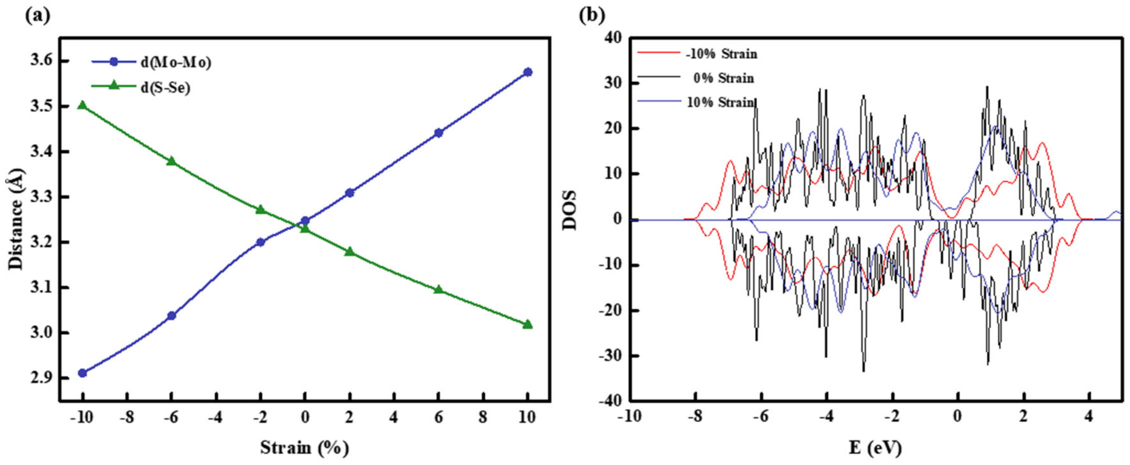

Figure S13 Effect of strain on Fe@MoSSe (a) structure and (b) density of state.

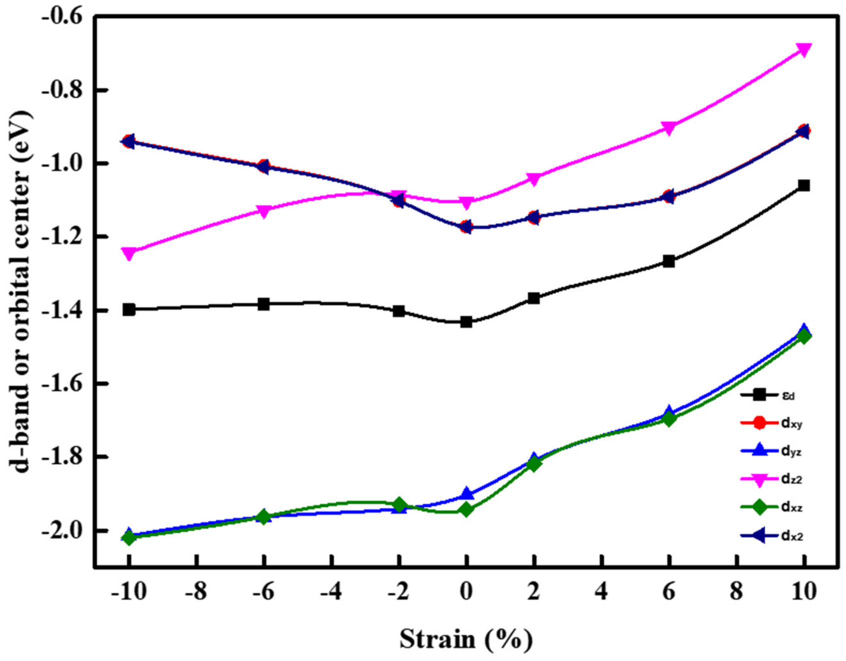

Figure S14 Effect of changing stress on different Fe@MoSSe d orbital.
